# A mobile application of breast cancer e-support program versus routine Care in the treatment of Chinese women with breast cancer undergoing chemotherapy: study protocol for a randomized controlled trial

**DOI:** 10.1186/s12885-017-3276-7

**Published:** 2017-04-26

**Authors:** Jiemin Zhu, Lyn Ebert, Xiangyu Liu, Sally Wai-Chi Chan

**Affiliations:** 1 Nursing Department, Medical College of Xiamen University, Xiangan Nan road, Xiangan District, Xiamen city, Fujian Province 361102 People’s Republic of China; 2 School of Nursing and Midwifery, Faculty of Health and Medicine, University of Newcastles, Richardson Wing, Callaghan University Drive, Callaghan, NSW 2308 Australia; 3 Hunan Cancer Hospital, the affiliated cancer hospital of Xiangya School of Medicine, Central South University, 283 Tongzipo Road, Yuelu District, Changsha, Hunan 410013 People’s Republic of China

**Keywords:** Breast cancer, Chemotherapy, Internet, Self-efficacy, Social support, Symptom distress, Quality of life, Psychological well-being

## Abstract

**Background:**

Women with breast cancer undergoing chemotherapy suffer from a number of symptoms and report receiving inadequate support from health care professionals. Innovative and easily accessible interventions are lacking. Breast Cancer e-Support is a mobile Application program (App) that provides patients with individually tailored information and a support group of peers and health care professionals. Breast Cancer e-Support aims to promote women’s self-efficacy, social support and symptom management, thus improving their quality of life and psychological well-being.

**Methods:**

A single-blinded, multi-centre, randomised, 6-month, parallel-group superiority design will be used. Based on Bandura’s self-efficacy theory and the social exchange theory, Breast Cancer e-Support has four modules: 1) a Learning forum; 2) a Discussion forum; 3) an Ask-the-Expert forum; and 4) a Personal Stories forum. Women with breast cancer (*n* = 108) who are commencing chemotherapy will be recruited from two university-affiliated hospitals in China. They will be randomly assigned to either control group that receives routine care or intervention group that receives routine care plus access to Breast Cancer e-Support program during their four cycles of chemotherapy. Self-efficacy, social support, symptom distress, quality of life, and anxiety and depression will be measured at baseline, then one week and 12 weeks post-intervention.

**Discussion:**

This is the first study of its kind in China to evaluate the use of a mobile application intervention with a rigorous research design and theoretical framework. This study will contribute to evidence regarding the effectiveness of a theory-based mobile application to support women with breast cancer undergoing chemotherapy. The results should provide a better understanding of the role of self-efficacy and social support in reducing symptom distress and of the credibility of using a theoretical framework to develop internet-based interventions. The results will provide evidence to support the implementation of an innovative and easily accessible intervention that enhances health outcomes.

**Trial registration:**

ACTRN: ACTRN12616000639426, Registered 17 May, 2016.

**Electronic supplementary material:**

The online version of this article (doi:10.1186/s12885-017-3276-7) contains supplementary material, which is available to authorized users.

## Background

Breast cancer is a major global public health problem. In China, breast cancer is growing as the most common cancer and the 6th leading cause of cancer-related death among women [1]. Adjuvant chemotherapy is a common treatment for invasive breast cancer in China, with approximately 81.4% of these women receiving chemotherapy [2]. The physical and psychological symptoms that occur after diagnosis and during chemotherapy can have a negative impact on the women’s quality of life (QoL) and psychological well-being [3]. Symptom management is therefore beneficial for women with breast cancer [4, 5], and reduced symptom distress is a crucial outcome of successful psychosocial interventions [6]. Symptom management requires that women understand appropriate management strategies and have appropriate levels of self-efficacy and social support to apply such strategies [7].

Evidence suggests that Chinese women with breast cancer receive inadequate support from health care professionals [8]. The increasing number of women with breast cancer [1], the insufficient financial commitment to health care from the government (5.5% of the GDP) [9, 10], and the shortage of oncology specialists [9] pose challenges to the feasibility of traditional face-to-face interventions. Internet-based interactive programs (IIPs) that enable health care professionals and patients to interact with each other via the internet to transmit health information and to offer and accept support can provide an innovative and easily accessible approach that can reach large groups of patients [11, 12].

Although preliminary evidence suggests that IIPs have had a positive impact in facilitating symptom management among women with breast cancer in Norway and the United States [6, 13], there is a worldwide paucity of rigorous trials that have evaluated the effectiveness of internet-based interventions for women with breast cancer [14]. In mainland China, there has been only limited investigation of the effectiveness of IIPs among Chinese women with breast cancer. Two such studies, published in Chinese, reported that IIPs improved symptom distress and self-efficacy [15] and decreased depression among women with breast cancer [16]. These two studies on IIPs in the Chinese population showed that the internet could be a useful tool for educating women about breast cancer. However, these two studies were not performed using randomised controlled trials (RCT). To the best of our knowledge, no RCTs have been conducted in Mainland China that evaluate the effects of an IIP for women with breast cancer undergoing chemotherapy.

In 2015, 50.3% of China’s population frequently used the internet [17], and 88.9% of these people access the internet by mobile devices [18]. With more women turning to mobile phone to search for information, mobile Applications (Apps) could provide a promising platform to apply IIP for women with breast cancer. However, a review of smartphone breast Apps reported the lack of evidence base and medical professional involvement in their development [19]. To improve women’s confidence in the use of Apps and further promote the implementation in health service, Mobasheri et al. [19] recommended that a robust framework, full authorship disclosure, and high quality clinical trials are needed to develop and test the effectiveness of Apps for women with breast cancer.

### The breast cancer e-support program (BCS)

The Breast Cancer e-Support program (BCS) is a mobile IIP application (App) for Chinese women with breast cancer who are undergoing chemotherapy. The BCS was developed using the theoretical framework [20], which incorporate Bandura’s self-efficacy theory [21] and the social exchange theory [22]. Appropriate levels of self-efficacy and social support are essential components of interventions to improve patients’ symptom management [23]. The BCS aims to enhance women’s self-efficacy and social support to promote their ability to manage the symptoms associated with breast cancer diagnosis and chemotherapy, thus improving their QoL and psychological well-being.

Self-efficacy, an individual’s perception of her ability to act effectively in a given situation, plays a crucial role in influencing a woman’s ability to manage her symptoms [21]. Self-efficacy comprises four main factors: direct mastery experiences, vicarious experiences, verbal persuasion and arousal state [21]. Direct mastery experiences involve previous personal accomplishments and successes. Vicarious experiences are gained by watching others achieve success in similar situations. Verbal persuasion comes from feedback and verbal cues from others. Finally, arousal states are defined as a person’s physiological state and their perception of that state.

Social support is defined as a combination of structural support and functional support [22]. Structural support consists of formal support from health care professionals and informal support from significant others. Functional support consists of exchange activities that take place among individuals, including emotional, instrumental, informational, and appraisal support. Access to a variety of types of functional support from different care providers, both health care professionals and significant others, is important for coping with stress [22].

**Fig. 1:**
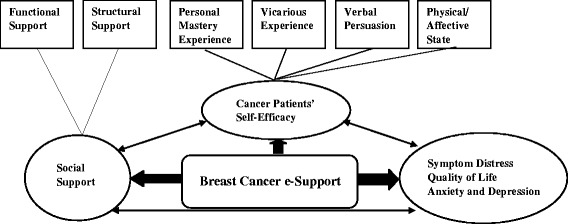
Theoretical framework of the Breast Cancer e-Support Program - adapted from Shorey et al. [21]

The BCS program is a multi-component intervention that includes a Learning forum, a Discussion forum, an Ask-the-Expert forum, and a Personal Stories forum. Four factors from the self-efficacy theory [21] and the functional and structural social support concepts from the social exchange theory [22] are incorporated into the BCS program. Regarding the self-efficacy theory, mastery experiences include the provision of symptom management knowledge in the Learning forum; vicarious experiences involve reading others’ personal stories in the Personal Stories forum and sharing experiences in the Discussion forum; verbal persuasion comes from feedback and verbal cues from peers and health care professionals in the Discussion forum and Ask-the-Expert forum; and the learning materials and sharing of experiences may modify women’s perceptions of their expected arousal states. In terms of social exchange theory, the Discussion and Ask-the-Expert forums are designed to build structural social networks and provide multiple types of functional support from peers and health care professionals. Figure 1 illustrates the theoretical framework of the BCS.

### Objectives

The objective of this RCT is to evaluate the effectiveness of the BCS program for Chinese women with breast cancer undergoing chemotherapy in terms of enhancing the women’s self-efficacy, social support and QoL, and reducing their symptom distress, anxiety and depression.

We hypothesise that compared with the control group at baseline, post-test 1 and post-test 2, the women with breast cancer who are undergoing chemotherapy in the experimental group will have the following significant differences:

1. Improved self-efficacy, social support and QoL;

2. Reduced symptom distress, anxiety and depression; and

3. Greater satisfaction with the health care received during chemotherapy.

### Trial design

A single-blinded, multicentre, randomised, controlled, parallel-group pre-test and repeated post-test superiority design will be used to investigate the effect of the BCS program for women with breast cancer who are undergoing chemotherapy.

## Methods

The study follows the SPIRIT 2013 Statement and the guidelines for the Standard Protocol of Clinical Trials [24, 25]. The study received ethic approval from the University of Newcastle in Australia and the two participating hospitals in P. R. China. The registration number with the Australian New Zealand Clinical Trials Registry is ACTRN12616000639426.

### Study setting

This study will be conducted in two university affiliated tertiary public hospitals in the People’s Republic of China. At each participating hospital, an average of 40 women with breast cancer per month receive chemotherapy; approximately 15 of these women have access to the internet. The usual treatment for women with breast cancer at these two participating hospital consists of four cycles of chemotherapy, with each cycle lasting 21 days.

### Eligibility criteria

Participants will be recruited on the day they commence chemotherapy. The inclusion criteria will be: (1) at least 18 years of age; (2) diagnosed with breast cancer within the past 3–8 weeks; (3) treated with chemotherapy at the study sites; (4) able to access the internet with a mobile phone; (5) cognitively and physically capable of participating and completing self-report questionnaires; (6) contactable via telephone or email for follow up; and (7) able to speak and read Mandarin. The exclusion criteria for women will be: (1) having a concurrent major physical illness and (2) having a chronic mental health problem.

### Intervention

#### BCS program

A research team at the School of Nursing and Midwifery at the University of Newcastle, Australia, developed the intervention, with technical assistance from the Suncco Internet Company in the People’s Republic of China. The BCS program is supported by the National Natural Science Foundation of China (71503219).

The Breast Cancer e-Support (BCS) program has a fixed structure that covers four cycles of chemotherapy, which last approximately 12 weeks. Data from previous studies suggest that IIPs provide the greatest benefits to women that have recently been diagnosed with breast cancer and are in the early stages of treatment [26], and that an 8-week online intervention is enough to produce changes in psychological outcomes [27]. Thus, a 12-week intervention is considered sufficient to test the effectiveness of the BCS. A pamphlet has been developed to provide guidance for downloading the BCS program from the website http://breastesupport.xmu.edu.cn/, and navigating the various sites within the BCS. It is up to the women to decide how often they access the BCS and how long they use the intervention.

**Fig. 2:**
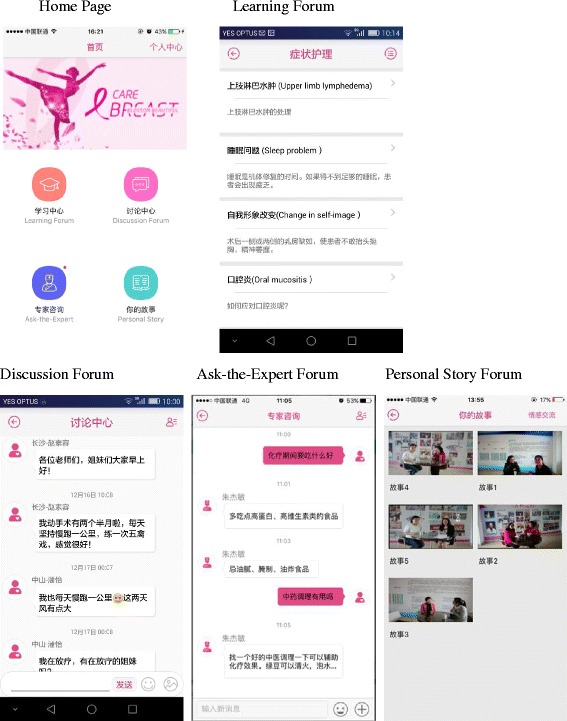
Screenshots of the BCS program home page and the four modules

The BCS has four modules: 1) a Learning forum; 2) a Discussion forum; 3) an Ask-the-Expert forum; and 4) a Personal Stories forum. These four modules are visually recognisable on the homepage of the BCS (see Fig. 2). The Learning forum provides 15 topics on symptoms management, including 1) nausea and vomiting management; 2) improving diet by making healthier food choices; 3) exercise and recovery; 4) how to deal with hand-foot syndrome; 5) how to deal with upper limber lymphoedema; 6) infection prevention and management; 7) fatigue management; 8) how to deal with depression and anxiety; 9) alopecia management; 10) oral mucositis management; 11) constipation management; 12) sleep management; 13) diarrhoea management; 14) how to deal with body changes; 15) bleeding and anaemia management. To create the 15 topics, detailed discussions with multidisciplinary Chinese oncology professionals were carried out. The Learning forum also provides basic information about breast cancer symptoms, diagnosis, stages, treatment options and community support service.

The Discussion forum and the Ask-the-Expert forum provide opportunities for social networking. In the Discussion forum, women will be invited to discuss symptom management topics and to share information and experience. In the Ask-the-Expert forum, health care professionals (a doctor and a senior nurse) will respond to health-related questions posed by individuals within 24 h. Only the individual that posed the question and the health care professionals will have access to the question and to the response. In the Personal stories forum, five recorded interviews with women who completed chemotherapy after breast cancer diagnosis and surgery will be available. Women with different stages of breast cancer, different ages, and different socio-economic statuses will be interviewed and recorded to provide role models for women with different needs. The moderator, an experienced health care professional, will moderate the online forum by reading all messages daily, facilitating online discussions, providing expert information when requested, and intervening appropriately if the messages posted are inappropriate or hostile.

#### Privacy

A member of the research team will help the women in the intervention group to register with the BCS program. The women will be initially assigned their mobile phone number as the user name; however, they can change their user names later by themselves. The women will submit their applications to the BCS program. Once the researcher approves applications through the App background management system, they women will set their own passwords. The user name will expire 12 weeks after activation. The participants will be informed that the researcher can access their profile.

#### Support

The researcher will offer personal training (averaging 30 min) to the experimental group to help its members download and install the App onto their mobile phones, demonstrate the usage of each module of the BCS, and help the women write an introductory message in the Discussion forum and ask a question in the Ask-the-Expert forum.

Technical assistance will be available via email or telephone from 8:00 am to 5:00 pm from Monday to Friday. At other times, users can email a technical assistant or leave a message on voicemail. A technical assistant will check emails and voicemail messages five days a week and respond to the women as soon as possible.

#### Fidelity of the intervention

Different strategies will be used to enhance the fidelity of the BCS, such as maintaining consistency in the delivery of the intervention and monitoring and reinforcing the women’s adherence to the intervention. To maintain consistency in the delivery of the intervention, the same researcher (ZJM) will organise the topics, give feedback and verbal cues, and provide encouragement and suggestions in the Discussion forum to the women regarding their progress with symptom management. The frequency and duration of the logins will be monitored to evaluate the women’s adherence. The women’s questions will be answered by health care professionals within 24 h to improve adherence to the BCS program.

#### Comparison

The women in both the experimental and control groups will receive routine care provided by the hospital. Routine care involves support from doctors and nurses during the two-day hospitalisation for each cycle of chemotherapy. Before treatment commences, a nurse will provide information on chemotherapy and possible side effects. There are currently no IIPs for education and social support provided by health care services at the study sites. The control group may freely use the internet to search for information about breast cancer. However, they will not have access to the BCS program.

### Outcome measures

#### Primary outcomes

##### Stanford Inventory of Cancer Patient Adjustment (SICPA)

Self-efficacy will be measured using the Stanford Inventory of Cancer Patient Adjustment (SICPA) [28]. The SICPA is a 38-item instrument assessing patients’ beliefs in their ability to manage cancer-related problems. Higher total scores equate to a higher level of self-efficacy. The SICPA has demonstrated good psychometric properties [28]. The SICPA has been translated into Chinese and validated in patients with cancer. The Chinese version of the SICPA has Cronbach’s alpha reliability coefficients of 0.95 [29].

##### Multidimensional Scale of Perceived Social Support (MSPSS)

Social support will be evaluated using the Multidimensional Scale of Perceived Social Support (MSPSS) [30]. The MSPSS is a 12-item self-report instrument assessing the respondent’s perceptions of support from three sources: family, friends and a significant other. A mean score greater than 5 indicates good support, a score of 3 to 5 indicates moderate support, and a score of less than 3 indicates poor social support. The original MSPSS has good reliability and validity [30]. The MSPSS has been translated into Chinese and validated in a Chinese population. The Chinese version of the MSPSS has adequate internal consistency (Cronbach’s α = 0.92) and construct validity [31].

### Secondary outcomes

#### M.D. Anderson symptom Inventory (MDASI)

Symptom distress will be assessed using the M.D. Anderson Symptom Inventory (MDASI) [32]. The MDASI consists of a 13-item symptom scale and a 6-item interference scale. A mean score of 1 to 4 indicates mild symptom distress, 5–6 indicates moderate symptom distress, and 7–10 indicates severe symptom distress. The original MDASI has demonstrated good reliability and validity [32]. The MDASI has been translated into Chinese and used in Chinese patients with cancer. The Chinese version of the MDASI has Cronbach’s alpha reliability coefficients of 0.74 and 0.88 for the symptom subscale and interference subscale, respectively [29].

#### Functional Assessment of cancer treatment-B (FACT-B, version 4)

Quality of life related to breast cancer will be measured using a Chinese version of the Functional Assessment of Cancer Treatment-B (FACT-B) [33]. FACT-B is a 37-item instrument assessing the impact of breast cancer and chemotherapy on dimensions of QoL. A higher total score means a better QoL. The English version of FACT-B has demonstrated good reliability and validity for women with breast cancer [34]. FACT-B has been translated into Chinese and has been widely used to assess the QoL of Chinese patients with different types of cancer. The Chinese version of FACT-B has a Cronbach’s alpha reliability coefficient of 0.87 for the entire scale [35].

#### Hospital anxiety and depression scale (HADS)

Anxiety and depression will be assessed using the Hospital Anxiety and Depression Scale (HADS) [36]. The HADS is a 14-item instrument assessing depression and anxiety in a medical setting. Higher total scores indicate greater distress. The original HADS has shown good reliability and validity in breast cancer survivors [37]. The HADS has been translated into Chinese and is widely used in the Chinese population with different types of health conditions. The Cronbach’s alpha coefficients of the Chinese version of HADS are 0.806 and 0.724 for the anxiety and depression subscales, respectively [3].

### Other outcomes

#### Satisfaction with care

Women’s satisfaction with their care during chemotherapy will be assessed with a six-item checklist developed for this study. Questions such as “Overall, how satisfied are you with the care so far?” will be rated on a five-point Likert scale, with 1 meaning “not at all” and 5 meaning “very much”. For each subscale, higher scores indicate greater satisfaction (Additional file 1).

#### Internet usage data for the BCS

The website contains a tracking system. Internet usage data, such as the frequency and duration of logins and the website activity of each module of the BCS, will be recorded and evaluated.

#### Demographic and clinical data

The data collected will include the women’s age, marital status, highest education level, employment status, monthly family income and BMI. Disease-related information will be provided by the hospital when the women are recruited; such information will include breast cancer stage, types of surgery, comorbidities and postoperative complications.

### Participant timeline

**Fig. 3:**
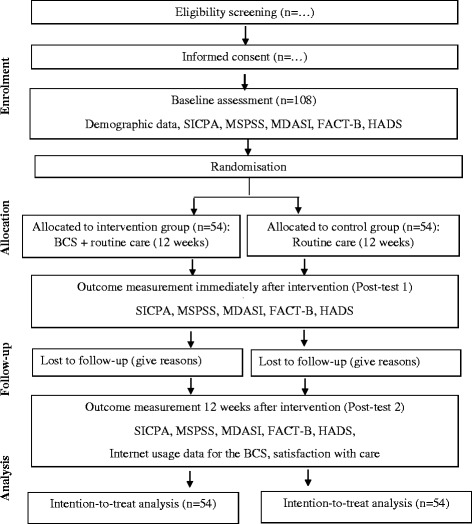
CONSORT flowchart of the study - adapted from Schulz et al. [38]. Note: SICPA: Stanford Inventory of Cancer Patient Adjustment; MSPSS: Multidimensional Scale of Perceived Social Support; MDASI: M.D. Anderson Symptom Inventory; FACT-B: Functional Assessment of Cancer Treatment-B; HADS: Hospital Anxiety and Depression Scale

This study began recruiting participants in May 2016, and the primary endpoints (baseline and post-test 1) and follow-up measure (post-test 2) are expected to be completed in February 2017. The Consolidated Standards of Reporting Trials (CONSORT) flowchart is presented in Fig. 3 [38].

### Sample size

The primary outcome of self-efficacy will be used to estimate the effect size based on power analysis [39]. A previous study on IIPs for women with breast cancer that measured QoL, social support, and health competence reported a medium effect size (0.46); however, the effect size for self-efficacy was not mentioned [40]. Similar research on psychoeducation to enhance the self-efficacy of Chinese patients with colorectal cancer also demonstrated a medium effect size of 0.60 for self-efficacy [29]. We estimate a 20% attrition rate based on a dropout rate of 10–15% in previous studies involving IIPs [27, 40]. To achieve a power of 0.80 at a 0.05 level of significance, a minimum of 108 women (54 in each group) are required for this study.

### Recruitment

Recruitment will take place before the women begin chemotherapy. The physician will introduce the BCS to women who meet the inclusion criteria and will provide the women with the contact details of the research assistant (RA). Women that have contacted the RA will be approached by the RA at the oncology unit to provide them with additional information regarding the BCS and to answer any questions that they might have. Women who verbally agree to participate will be followed up to obtain their written consent. The enrolment period is expected to extend over a 6-month period. Data will be collected before randomisation (baseline), immediately after the intervention (post-test 1), and 12 weeks after the intervention (post-test 2). The women will be rewarded with a small gift (equal to RMB 30) for each questionnaire completed.

### Randomisation

For each hospital, the Research Randomizer [41] will be used to generate a random set of 27 unique, non-duplicating numbers from 1 to 54. Different coloured slips (pinks slips for randomly generated numbers, white slips for all other numbers) will be put into an opaque envelope. The participants that pick a pink slip from the opaque envelope will be assigned to the experimental group; the others will be assigned to the control group. The women in the intervention group will have access to the BCS program and receive routine care while undergoing treatment. The women in the control group will receive routine care during treatment. The health care providers and the RA collecting the data will be blinded to the participants’ group allocation.

### Data analysis

All outcome measures will be analysed using IBM SPSS Statistics 21.0 [42]. An intention-to-treat analysis will be adopted to manage missing data. The baseline differences between the intervention and control groups will be assessed using the chi-square (χ^2^) test for binary demographic data and the independent sample t-test for continuous variables. Adjusted for possible confounding factors of demographic variables, the repeated measures triply MANCOVA will be conducted to determine whether the BCS intervention is effective in improving self-efficacy, social support and QoL and for reducing symptom distress, anxiety and depression across three time points of data collection (baseline, post-test 1 and post-test 2). The independent t-test will be used to compare how satisfied the experimental group and the control group are with the care that they received during chemotherapy 12 weeks after intervention.

### Ethics, consent and permissions

The ethics approval for this study has been obtained from the Human Research Ethics Officer at the University and the participating hospitals. This study will adhere to ethical standards for the whole procedure. Women will not be deprived of any treatment and routine care, and there is no potential rick or harm by participating in this program. Women will be assured that participation in this study is voluntary and they can withdraw from the study at any point of time without any effect on their treatment. The consent form will be obtained and all data will be kept confidential and anonymous.

## Discussion

This study will contribute to evidence on the effectiveness of using a theory-based BCS program to support women as they cope with the unique challenges of breast cancer and chemotherapy. The results of this study will provide a better understanding of the role of self-efficacy and social support in reducing symptom distress and the credibility of using a theoretical framework to develop a BCS intervention. Such knowledge may help to advance research regarding the use of internet-based interactive methods to promote women’s symptom self-management, thus improving women’s QoL and psychological well-being.

If the BCS program is effective, it could be offered by health care professionals as part of the routine treatment to enhance health outcomes for women with breast cancer who are undergoing treatment in China. The knowledge gained from this study could be used to plan a culturally appropriate BCS program for other cancer patients. If the BCS program is effective, this study will provide evidence to support the need to train staff in e-health and to allocate resources for developing e-health to further advance this effort. To the best of the researchers’ knowledge, this is the first study of its kind that evaluates a BCS intervention using a rigorous research design and a theoretical framework in China.

## Additional file

Additional file 1: Satisfaction Evaluation Questionnaire. (DOCX 23 kb)

## Abbreviations

APP: Mobile application
BCS: Breast Cancer e-Support
FACT-B: Functional Assessment of Cancer Treatment-B
HADS: Hospital Anxiety and Depression Scale
MDASI: M.D. Anderson Symptom Inventory
MSPSS: Multidimensional Scale of Perceived Social Support
RA: Research assistant
RCT: Randomised controlled trial
SICPA: Stanford Inventory of Cancer Patient Adjustment
