# Supplementary material for: A mobile application of breast cancer e-support program versus routine Care in the treatment of Chinese women with breast cancer undergoing chemotherapy: study protocol for a randomized controlled trial
Source: BMC Cancer. 2017 Apr 26;17:291. doi: 10.1186/s12885-017-3276-7 (PMC5406970; doi:10.1186/s12885-017-3276-7)
Supplement: Additional file 1: — Satisfaction Evaluation Questionnaire. (DOCX 23 kb) [file 12885_2017_3276_MOESM1_ESM.docx]

**Satisfaction Evaluation Questionnaire**

Instruction: Listed below are a series of your evaluation of this program. Read each item carefully and circle the number (ranging from “0” to “5” which best present your appraisal of this program. Make sure your evaluating ratings accurately reflect your judgement of this program.

①not at all ② Not much ③ Neutral ④ Somewhat ⑤ very much

| 1. Did you find the care provided to you beneficial especially in enhancing your confidence? | ① ② ③ ④ ⑤ |
| --- | --- |
| 2. Did you find the care provided to you useful in improving your social support? | ① ② ③ ④ ⑤ |
| 3. Did you find the care provided to you useful in improving your symptom management behaviours? | ① ② ③ ④ ⑤ |
| 4. Did you find the care provided to you useful in improving your quality of life? | ① ② ③ ④ ⑤ |
| 5. Did you find the care provided to you useful in improving your mood and decreasing your negative feeling? | ① ② ③ ④ ⑤ |
| 6. Overall, how satisfied are you with the care so far? | ① ② ③ ④ ⑤ |

**患者满意度调查**

说明:请圈出你对医院服务的评价

① 极不同意 ② 稍不同意 ③ 中立 ④ 稍同意 ⑤ 非常同意

| 1. 您有没有发现护理以及服务可以增强你症状管理的自信心呢? | ① ② ③ ④ ⑤ |
| --- | --- |
| 2. 您有没有发现护理以及服务可以增强你寻找帮助的能力呢? | ① ② ③ ④ ⑤ |
| 3. 您有没有发现护理以及服务可以增强你症状管理的能力呢? | ① ② ③ ④ ⑤ |
| 4. 您有没有发现护理以及服务可以提高您的生活质量呢? | ① ② ③ ④ ⑤ |
| 5. 您有没有发现护理以及服务可以使您心情舒畅一点, 可以减少您的负面情绪呢? | ① ② ③ ④ ⑤ |
| 6. 总的来说, 您对护理以及服务满意吗? | ① ② ③ ④ ⑤ |
